# Supplementary material for: The Mysterious Amurian Grig Paracyphoderris erebeus Storozhenko, 1980 (Orthoptera: Prophalangopsidae): New Data on Its Distribution, Ecology and Biology
Source: Insects. 2023 Sep 27;14(10):789. doi: 10.3390/insects14100789 (PMC10607618; doi:10.3390/insects14100789)
Supplement: Supplementary file 1 [file insects-14-00789-s001.zip › insects-2567016-supplementary.pdf]

Table S1. Geographic coordinates of known records of *Paracyphocerris erebeus* in its range.

Region	Locality	Geographic coordinates	Altitude, m	Date	Specimens	Collector	Natural Reserve
Khabarovsk	**Myaochan Ridge, 40 km NW Komsomolsk-on-Amur, settlement Tikhy**	**50.7458 N/ 136.5114 E**	**650**	**23.08.1976**	**1♂, 2♀**	**V.A. Mutin**	
	same locality			5.07.1997	2 nymphs	V.A. Mutin	
	Myaochan Ridge, Levaya Silinka River	50.7403 N/ 136.4794 E	550	25.06.1982	1♀, 3 nymphs	V.A. Mutin	
	Myaochan Ridge, settlement Gorny, elfin woodland	50.7686 N/ 136.3922 E	650	29.08.1983	2♀, 10 nymphs	S.Yu. Storozhenko	
	same locality			10.07.1986	1♂	V.Ya. Nebaikin	
	same locality			19.06.1997	1♂	V.A. Mutin	
	Myaochan Ridge, 3 km NE settlement Gorny, alder woodland	50.7803 N/ 136.4764 E	600	16–17.07.2011	2♀	E.S. Koshkin	
	Myaochan Ridge, 10 km SW settlement Gorny, spruce forest	50.7228 N/ 136.3128 E		8.06.1984	1 nymph	V.N. Makarkin	
	same locality		800	28.05.2005	1♀, 1 nymph	M.Yu. Proshch alykin	
	same locality			29.06.2005	2 nymphs	V.A. Mutin	
	same locality		500	29.06.2005	1♂	A.S. Lelej	
	Badzhalsky Ridge, Gerbi River basin, upper stream of Omot Makit River	50.5092 N/ 134.5458 E	950–2,100	5–19.07.1997	12♂, 6♀, 3 nymphs	A.V. Plutenko	
	Dusse-Alin Ridge, Bureika River, subalpine belt	51.1061 N/ 133.5989 E	900–1,100	15–20.06.2009	2♂	E.S. Koshkin	Bureinsky
	Dusse-Alin Ridge, 5 km below the confluence of the Pravaya and Levaya Bureya Rivers, subalpine belt	51.6292 N/ 134.2578 E	900–1,100	20.07.2000	1♀, 1 nymph	A.G. Blyummer	Bureinsky
	Dusse-Alin Ridge, 3 km below the confluence of the Pravaya and Levaya Bureya Rivers,	51.6417 N/ 134.2769 E	570	6.06.2011	1♂	E.S. Koshkin	Bureinsky
	Dusse-Alin Ridge, upper stream of Levaya Bureya River, vicinity of Korbokhon Lake	52.0264 N/ 135.0728 E	1,200	27.06.2011	1♂	E.S. Koshkin	Bureinsky
	Dusse-Alin Ridge, upper stream of Levaya Bureya River, vicinity of Medvezhy Lake,	52.0886 N/ 135.0117 E	1,615	2.07.2011	1♂	E.S. Koshkin	Bureinsky
	Dusse-Alin Ridge, upper stream of Levaya Bureya River, pass between the lakes Medvezhy and Gornoe	52.0803 N/ 135.0208 E	1,755	2.07.2011	1♂, 1 nymph	E.S. Koshkin	Bureinsky

	Bureinsky Ridge, Urma River basin, upper stream of Pravy Omot River, scree	50.4222 N/ 134.2072 E	1,600–1,650	29.06.1978	1♂	A.A. Nazarenko	
	Aezop Ridge, subalpine belt	52.5661 N, 134.0369’ E	1,400	10.07.1978	2♂	E.V. Novomodnyi	
Jewish Autonomous Region	Bastak Nature Reserve, Bydyr Mountain	49.2108 N/ 133.0579 E	1,200	29.05.2014	1 nymph	A. Averin	Bastak
	same locality			17.06.2022	1♂, 1 nymph	A. Averin	Bastak

In bold — the type locality
